# Psychological approach to successful ageing predicts future quality of life in older adults

**DOI:** 10.1186/1477-7525-9-13

**Published:** 2011-03-09

**Authors:** Ann Bowling, Steve Iliffe

**Affiliations:** 1 Faculty of Health and Social Care, St George's, University of London and Kingston University, St George's, University of London, Cranmer Terrace, London SW17 ORE, UK; 2 Department of Primary Care and Population Sciences, University College London, Hampstead Campus, London NW3 2PF, UK

## Abstract

**Background:**

Public policies aim to promote well-being, and ultimately the quality of later life. Positive perspectives of ageing are underpinned by a range of appraoches to successful ageing. This study aimed to investigate whether baseline biological, psychological and social aproaches to successful ageing predicted future QoL.

**Methods:**

Postal follow-up in 2007/8 of a national random sample of 999 people aged 65 and over in 1999/2000. Of 496 valid addresses of survivors at follow-up, the follow-up response rate was 58% (287). Measures of the different concepts of successful ageing were constructed using baseline indicators. They were assessed for their ability to independently predict quality of life at follow-up.

**Results:**

Few respondents achieved *all *good scores within each of the approaches to successful ageing. Each approach was associated with follow-up QoL when their scores were analysed continuously. The biomedical (health) approach failed to achieve significance when the traditional dichotomous cut-off point for successfully aged (full health), or not (less than full health), was used. In multiple regression analyses of the relative predictive ability of each approach, only the psychological approach (perceived self-efficacy and optimism) retained significance.

**Conclusion:**

Only the psychological approach to successful ageing independently predicted QoL at follow-up. Successful ageing is not only about the maintenance of health, but about maximising one's psychological resources, namely self-efficacy and resilience. Increasing use of preventive care, better medical management of morbidity, and changing lifestyles in older people may have beneficial effects on health and longevity, but may not improve their QoL. Adding years to life and life to years may require two distinct and different approaches, one physical and the other psychological. Follow-up health status, number of supporters and social activities, and self-rated active ageing also significantly predicted QoL at follow-up. The longitudinal sample bias towards healthy survivors is likely to underestimate these results.

## Background

The current generation of ageing adults expects to age well, and to maintain their general well-being and, ultimately, enhance the quality of later life. Most people aged 50 and 65 and more rate themselves as ageing well, or successfully, and few rate as high their chances of becoming housebound, losing their memory or entering a nursing home [1,2]. These positive perspectives reflect a shift away from a predominantly pathological perspective of later life, which exaggerated the extent to which chronic ill-health could be attributed to ageing, and which largely ignored the heterogeneity of the older population. A more positive view of old age sees it as a period of opportunity and well-being, with retention, or development, of the psychological resources to cope with life's challenges [3]. This coincides with world-wide policy interest in the promotion of physical and mental well-being in populations, and the compression of morbidity into fewer years of later life, driven by concerns about increasing expenditure on health and social care in an ageing society. Although there is an awareness that well-being has no clearly defined opposite, and that it is more than the absence of 'ill-being', there are no agreed definitions, other than that it is a 'good thing' [4,5]. Policy guidance, including that in the UK, prefers to focus on specific aspects of well-being that are potentially amenable to known interventions, including physical activity (e.g. exercise) to maintain mental and physical functioning, hence well-being [6], rather than as a dynamic, multi-faceted state which includes more complex subjective, social, and psychological dimensions. There are however exceptions to such reductionist views [7,8]. For example, NHS Scotland (2006) [8] defined the state of mental well-being broadly, encompassing subjective and psychological feelings of life satisfaction, optimism, self-esteem, mastery and feeling in control, having a purpose in life, a sense of belonging and support. This is more consistent with the long tradition of social research on general well-being, dating back to the 1950s [1].

The current, international policy focus on promotion of well-being has stimulated interest in quality of life (QoL) as an outcome indicator. QoL has long been used as an outcome measure in the evaluation of a diverse range of health and social care interventions. It is a multi-faceted, concept, encompassing macro societal and socio-demographic influences and also micro concerns, such as individuals' experiences, social circumstances, health, values and perceptions [1]. As it is subjective, it needs grounding in people's own values and perceptions.

Much of the focus on how to enhance the quality of later life has been on the achievement of successful ageing, by promoting different approaches, ranging from biomedical, as in the MacArthur Studies of Successful Aging [9-11], to broader social,-psychological and lay-based approaches [3,12]. These overlap with concepts of 'active ageing' [13]. The criteria necessary for achieving successful ageing, described in the literature, can be grouped into five approaches: biological (i.e. 'health'), broader biological (i.e. health and social engagement), social, psychological and lay. These have have been reviewed in detail in a cross-disciplinary systematic review of successful ageing [3], and their construction for the research reported here is summarised next (the measurement scales are described later under Methods):

• **Biomedical (i.e. health)**: Comprised summing of: having diagnosed, chronic medical conditions (actual number reported); ability to perform activities of daily living (ADL) (originally no/little difficulty was originally scored <10, with the remainder scoring a range of levels of difficulty); psychiatric morbidity measured using the General Health Questionnaire-12 (GHQ-12) (original caseness was scored as 5 or more, with no problems as 0-4).

• **Broader biomedical (i.e health and social engagement)**: Comprised summing of the above plus number of different social activities engaged in during past month (3+), as an index of social engagement.

• **Social functioning**: Comprised summing of number of different social activities engaged in during past month, frequency of social contacts, number of helpers/supporters.

• **Psychological resources**: Comprised summing of self-efficacy score (best score was less than an original score of 11), best optimism score (of less than an original score of 6), plus GHQ-12 items on sense of purpose: playing useful part; coping: facing up to problems, overcoming difficulties; self-esteem: feels has self-confidence and has self-worth.

• **Lay**: Comprised summing of the above (note: GHQ-12 items were counted once only given their overlap across models, to avoid singularity being violated by double summing), plus gross annual income and perceived social capital [rating of area facilities (e.g. transport, closeness to shops, services), area problems (e.g. crime, vandalism, graffiti, speed and volume of traffic, air quality), somewhere nice to go for a walk, feels safe walking alone during the day or night].

Biological (or health) approaches to achieve successful ageing have been defined as the avoidance of disease and risk factors, maintenance of physical and cognitive functioning and active engagement with life [9]. Some biological appraoches are broader, also including numbers of different social activities engaged in during past month (i.e. health and social engagement). Current social approaches include maintenance of high levels of social activity, interaction and participation [14]; and psychological approaches emphasise psychological resources for coping with the challenges of ageing over time (e.g. perceived self-efficacy, control over life, ability to compensate for declining abilities [15,16]. While biological approaches have been the most often investigated [3], broader approaches, including psycho-social factors accord more closely with lay views of successful ageing [2] that include income and environmental quality and safety. In cross-sectional analyses such broader biological approaches are also associated with people's self-rated quality of life [12]. These have been reviewed in depth by Bowling [3].

In earlier work on alternate criteria of successful ageing, we reported that broader approaches predicted self-rated QoL more powerfully than unidimensional approaches, and should be used to evaluate the outcomes of health promotion interventions in the older population [12]. This paper investigates the predictive ability of these different biological, psychological and social approaches of successful ageing on QoL over time, using a national random sample of people aged 65 and over who were followed up 7-8 years later.

## Methods

A postal follow-up survey of community-dwelling people aged 65 and over who had responded to four face-to-face interview surveys about QoL during 1999/2000. The sample was derived from four quarterly Office for National Statistics Omnibus Surveys during 2000-1, sampled quarterly from a small user postcode sampling frame across Britain, with geographic and socio-economic stratification.

Omnibus Survey respondents aged 65 and over were asked whether they would be willing to be re-interviewed by ONS interviewers for our module on QoL. Those who consented were re-interviewed two months later. Of the sample of 1,299 eligible respondents sifted by Omnibus Survey staff, the overall response rate was 77% (999), 19% refused and 4% were not contactable. The characteristics of the baseline sample were broadly representative of people aged 65 and over living at home in Britain and have been reported in detail [1].

After removing the addresses of non-survivors identified from flagging checks at NHS Central Registry), survivors aged 65 and over at baseline were mailed a further postal questionnaire in 2007-8 (n- = 553), containing measures of QoL, active ageing, health, psych-social and economic circumstances. Of these 553 mailings, relatives replied and informed us that a further five sample members had died, and the Royal Mail returned a further 52 envelopes (9% of the 553 mailings) as 'person not at/unknown at that address. Apart from sample flagging at ONS Central Registry, although there will be a time lag before revisions are received, logged and released, all baseline respondents were also given a Freepost card on which to notify us of changes of address. As the follow-up study was postal, there was no opportunity for interviewers to approach neighbours for information about moves.

A total of 287 completed questionnaires were returned by respondents. The raw response rate at final follow-up, then, was 287 out of 553 mailed: 52%. The response was 52% if deaths were removed from the denominator (302/553 minus 5 deaths = 287/548). The valid response rate of 287 questionnaires returned out of 496 valid addresses (removing both 5 deaths and 52 untraced respondents from the denominator = base = 496) was 58%.

Sample attrition is inevitable in longitudinal surveys of older adults, where the most vulnerable and ill members of the sample will have died, leaving the healthiest sample members. The follow-up sample was, by definition, a sample of survivors. As the main source of non-response was death, baseline characteristics of survivors and deceased sample members by follow-up were compared. These confirmed the expectation that the deceased respondents were more likely than survivors to be older, male, and less likely to rate their health optimally. For example, of those who died by follow-up: 58% (133) were in the oldest age group 75+, compared with 37% (283) of survivors (Chi-square:55.260, 3df, p = 0.001); 58% (132) were male, compared with 45% (343) of survivors (Chi-square: 12.139, 1df, p = 0.001); and 59% (135) had rated their health at baseline as 'Excellent/Very good', compared with 77% (590) of survivors (Chi-square: 29.338, 1df, p = 0.001). Hence, the results presented here need to be interpreted with caution, given the healthier survivor bias.

The sample (287) was initially weighted by ONS to correct for the unequal probability of small households (in which people aged 65 and over usually live) being included in the sample and this increased the effective sample size to n = 302.

The baseline study was granted ethical committee consent by London MREC and ONS Omnibus ethics committee; the follow-up study was granted ethnical committee consent to proceed by University College London Research Ethics Committee, and registered with UCL Clinical Governance.

### Measures

QoL was the dependent variable, measured using the follow-up QoL measure: the Older People's Quality of Life Questionnaire (OPQOL). The OPQOL was designed to be multi-dimensional, and was developed directly from older people's views on the main components of QoL [1,17]. It had 32 items with 5-point Likert scales ('Strongly Agree' to 'Strongly Disagree'), representing: life overall (4 items), health (4 items), social relationships and participation (7 items), independence, control over life, freedom (5 items), area: home and neighbourhood (4 items), psychological and emotional well-being (4 items), financial circumstances (4 items). Items are scored with higher scores equalling higher QoL; the scale ranges from 32 to 160; cut off points indicate levels of quality of life [17]. The OPQOL had good psychometric properties when tested on independent population and ethnically diverse sample surveys in Britain; it had better reliability and validity overall than other QoL instruments [17]. Cronbach's alphas for the OPQOL was α 0.901, and thus satisfied the α: 0.70 < 0.90 threshold for internal consistency: α: 0.901 [17].

The variables selected for the construction of the alternate approaches to successful ageing (see Box 1) were dichotomised into 'good' and 'not good' scores. The number of good scores for each item included (see below) was used to represent successful ageing for the different approaches. Both numbers of good scores within each approach, as well as traditional cut-off points (achieving all or mostly good scores for the indicators included within an approach [9]) were analysed. The biomedical approach was thus a sum of positive responses, indicating no problems to physical and psychological health variables (diagnosed, chronic medical conditions, activities of daily living, no psychological morbidity using the General Health Questionnaire-12 [18]). The social functioning approach comprised summing of: number of different social activities engaged in during past month, frequency of social contacts, and number of helpers and supporters. The psychological resources approach involved summing of positive self-efficacy score, best optimism score, and positive responses to General Health Questionnaire-12 (GHQ-12) [18] items measuring sense of purpose, coping, self-confidence and self-worth (these items were removed when the biological, social and psychological approaches were entered together in a multivariate analyses to examine their independent predictive ability). In addition to the GHQ-12 [18], the psycho-social variables above were measured with validated scales of social support [19], perceived neighbourhood environment [20], self-efficacy [21], optimism-pessimism [22], and items measuring social activities, loneliness, life expectations, risk perceptions, and social comparisons. Physical health and functioning was measured with Townsend's [23] physical functioning [activities of daily living (ADL)] scale; self-rated health; and diagnosed medical conditions. Standard socio-demographic and economic items were also included in the questionnaire. These included age, sex, socio-economic status (NS-SEC), housing tenure, gross annual income, age left full-time education, highest education qualification, household size, and marital status. Indicators of successful ageing were selected after examination of the literature [3,12].

### Statistical analysis

The OPQOL was selected on the basis of its multi-dimensionality as the outcome indicator against which to test the independent predictive ability of the approaches to successful ageing. The OPQOL was developed from open-ended responses to questions about quality of life at baseline, and tested in the follow-up survey. Thus there was thus no baseline multidimensional measure of OPQOL.

Item non-response was minimal at baseline. The range of baseline item-non-response was16-21 out of the 999 respondents. This was due to the baseline study being a face-to-face interview survey, conducted by trained interviewers from the office of National Statistics (and whose training emphasised the importance of item response). The follow-up item response was less good as the mode was self-completion (postal). The range of follow-up item non-response was 55-58 out of the 302 weighted sample (287 raw sample size)

Univariate analyses included frequency distributions, Spearman's rho correlations, means, and chi-square tests. The Spearman rank correlation coefficient is calculated on occasions when it is not possible to give actual values to variables, but only to assign a rank order to instances of each variable. Sex was coded in rank order (0,1) it was therefore legitimate to use this method.

Linear multiple regression analysis was used for model comparison in relation to quality of life outcomes (after checks for multicollinearity). The ability of theoretically relevant variables to independently predict successful ageing classifications was tested. There are inconsistent associations in the literature between socio-demographic variables and indicators of well-being, including quality of life, and these were included last (to control for their effects) [1]. A hierarchical approach was used, with entry of independent variables in theoretical order of importance. The level for statistical significance was set at 0.05. Item non-response was small, although cumulative.

The scales and items included in the baseline measures of successful ageing, were conceptually distinct from the lay-based 'OPQOL' at follow-up. They did not over-correlate by more than 0.60, and satisfied criteria for multicollinearity. For example, the baseline measures were more objective indicators (e.g. number of chronic conditions to number of social contacts). In contrast, the follow-up OPQOL was subjective and contained evaluative items (e.g. feelings of needing (more) companionship).

The multiple regression analysis was limited to testing the biomedical (i.e. health), social and psychological approaches, as independent predictors of quality of life, as they overlapped in content with the broader biomedical (i.e. health and social engagement) and the multidisciplinary lay approaches.

## Results

### Characteristics of sample

The baseline sample was evenly divided between men and women, just under two thirds, were aged 65 < 74, and the remainder were aged 75 and over; most were married although over a quarter were widowed; and a third lived alone; Less than half had an income of £7,280 or more. The vast majority of respondents were white, as would be expected in a national sample of people aged 65 and over [1]. At follow-up, 17% (47) of the sample were aged 65 < 75; the remainder were all aged 75 and over. Over half of the sample comprised women (54%, 152). In addition, 49% (138) were married or cohabiting compared with being single or widowed; 49% (137) lived alone, rather than with others, and 85% (239) were owner-occupiers.

### Quality of life

OPQOL scores at follow-up were slightly positively skewed: 7% (17) scored as QoL as bad as can be (<11) and 12% (29) scored at the most optimum QoL end of the scale (140+). The mean OPQOL score was 121.385; standard deviation 14.048 (scale range 32-160, with higher scores equating with better QoL). The QoL sub-scales on which respondents scored most positively were home and neighbourhood, followed by psychological well-being and outlook (36% (106) and 30% (87) respectively scored 'QoL as good as can be'). The areas that they scored worst on were health and functioning and financial circumstances (21% (58) and 15% (45) respectively scored 'QoL as bad as can be'). This may be expected with the decline in health and financial reserves that often accompany older age. These areas also had the lowest mean sub-scale scores. There were no significant differences in mean score or subscale scores and age or sex of respondents.

### Successful ageing

Approaches to conceptualising successful ageing are traditionally constructed with dichotomous cut-off points (successfully aged, or not), with the requirement that, to be categorised as successfully aged, individuals should have met the criteria for successful ageing on each indicator included [9]. For this study, as stated earlier, both numbers of good scores within each approach, as well as traditional, dichotomous cut-off points indicating success were analysed. At both baseline and follow-up, the sample distributions were skewed positively towards people achieving higher numbers of good scores within all except the lay approach to successful ageing (which had a normal distribution). However, few achieved *all *good scores within each approach, indicating that traditional approaches are unrealistic as they exclude most people.

Table 1 shows the associations between baseline approaches to successful ageing, using traditional cut-offs for success (all or mostly good scores on each indicator in the approach), and follow-up OPQOL. The smaller numbers in the successfully aged groups are shown. Only the narrow biomedical (health) approach failed to achieve statistical significance at the 0.05 level with OPQOL categories.

**Table 1:** Baseline successful ageing+ by follow-up OPQOL++

**Successful ageing+++**:	**OPQOL: Quality of life is**:		
	**So bad could not be worse scores <99**	**Middle scores 100-119**	**So good could not be better scores 120+**
	**% (n)**	**% (n)**	**% (n)**

**Successful ageing biomedical (health)**			
Not successfully aged	81 (13)	70 (64)	60 (75)ns
Successfully aged on all 3/3 indicators	19 (3)	30 (27)	40 (50)

**Successful ageing broader biomedical (health and social engagement)**			
Not successfully aged	88 (14)	73 (66)	62 (77)*
Successfully aged on all 4/4 indicators	13 (2)	28 (25)	38 (48)

**Successful ageing psychological**			
Not successfully aged	100 (16)	85 (78)	72 (90)**
Successfully aged on all 7/7 indicators	--- ---	15 (14)	28 (35)

**Successful ageing social**			
Not successfully aged	81 (13)	57 (53)	38 (47)***
Successfully aged on all 3/3 indicators	19 (3)	43 (40)	62 (78)

**Successful ageing lay**			
Not successfully aged (<10)	100 (13)	81 (70)	50 (61)***
Successfully aged on 10-13 indicators	--- ---	19 (16)	50 (60)

**No. of responders**	**13-14**	**91-93**	**125**

NS not statistically significant using Chi-square tests at least at 0.050; * p < 0.05; ** p < 0.01; *** p < 0.001; Caution in interpretation is required where there are less then 5 counts per cell

+Recoded baseline scores; ++OPQOL scores grouped at follow-up

*+++ Biomedical (health): sum of (1 problem, 0 no problem) no diagnosed, chronic medical conditions, no problems with activities of daily living, no psychiatric morbidity (GHQ-12 with 5+ cut-off); broader biomedical model (health and social engagement): sum of: the above plus number of different social activities engaged in during past month (3+), as an index of social engagement; social functioning model: sum number of different social activities engaged in during past month (as above 3+), frequency of social contacts score (1-8), helped/supported in all 5 areas of life asked about.; psychological resources model: sum of self-efficacy score (best <11), best optimism score (<6), plus best ratings on single GHQ items (3, 6, 8, 10, 11) on: sense of purpose: playing useful part; coping: facing up to problems, overcoming difficulties; self-esteem: feels has self-confidence, has self-worth. Lay model: sum of all the above (note: duplicated items between above models counted once) plus gross annual income (>£7280), and optimal perceived social capital scores (ratings of area facilities, e.g. transport, closeness to shops, services;, area problems, e.g. crime, vandalism, graffiti, speed and volume of traffic, air quality;, somewhere nice to go for a walk, feels safe walking alone during the day or night)*.

Continuous scores for the measures of successful ageing were also analysed in relation to OPQOL scores. All approaches were then significantly associated with OPQOL, indicating that traditionally used cut-offs in approaches to successful ageing are less sensitive to quality of life (Table 2). The table also shows that follow-up social and psychological variables (except coping) were associated with OPQOL scores. Age but not sex was also associated with OPQOL, as was socio-economic status. The final column displays the correlations for those who had died between baseline interview and follow-up. Each approach to successful ageing, except the full and reduced psychological approach, was significantly correlated with mortality.

**Table 2:** Spearman's correlations with baseline successful ageing (continuous scores)

**Baseline**:	**OPQOL total score at follow-up 32 items**:	Died between baseline and follow-up interview
Successful ageing biomedical (health) raw score	0.361**	-0.159**

Successful ageing broader biomedical (health and social engagement) raw score	0.401**	-0.138**

Successful ageing psychological raw score	0.337**	-0.032 ns
*Successful ageing psychological model raw score reduced - self-efficacy and optimism only, minus GHQ items on self worth/confidence*	0.206**	-0.026 ns

Successful ageing social raw score	0.315**	-0.157**

Successful ageing lay raw score	0.489**	-0.138**

**Follow-up**:		

Self-rated health status - Excellent to Poor Likert scale	0.480**	NA

ADL raw score	-0.507**	NA

No. of people who comfort/support	0.397**	NA

No. of different social activities in last month	0.537**	NA

Self-rated ageing actively	-0.582**	NA

Efficacy- can handle whatever comes Likert scale	0.373**	NA

Efficacy: Can solve most problems- Likert scale	0.365**	NA

Coping methods - has identified method/none	-0.034 ns	NA

**Socio-demographic/economic**:		

Age - continuous	-0.227**	0.229**

Sex	0.088 ns	0.110**

NS-SEC classes	0.194**	-0.031 ns

Married/not+	0.148*	0.060 ns

ns not statistically significant at least at 0.050;* 0.05; ** p < 0.01 using Chi-square tests NA not applicable; + follow-up status married for OPQOL column and baseline married status for mortality column

### Multivariable analyses

It was not possible to enter all five approaches to successful ageing into a single linear multiple regression analysis, due to overlap between their items, compromising their independence. First, in order to examine their contribution to explained variation in OPQOL scores, each approach to successful ageing was entered singly into separate multiple regressions of predictors of OPQOL, along with the same follow-up variables. Results were similar for each approach to successful ageing, when entered separately, and each was highly significant. The proportions of explained variance in OPQOL scores were: 56% for biomedical (health) (Adjusted R^2 ^0.564; p = 0.0001); 57% for broader biomedical (health and social engagement) (Adjusted R^2 ^0.567; p = 0.0001); 58% for psychological (Adjusted R^2 ^0.576; p = 0.0001); 57% for social (Adjusted R^2 ^0.571; p = 0.0001); 60% for the lay approach (multidimensional incorporating each model (Adjusted R^2 ^0.598; p = 0.0001).

In order to assess their independent, relative contribution to OPQOL scores, the successful ageing approaches with independent (non-overlapping) items were entered into a single multiple regression together, along with follow-up items. The approaches entered together were the biomedical (health), the psychological (minus the items which overlapped with the GHQ in the biological approach) and the social approach to successful ageing. Table 3 shows the full regression model and Table 4 shows the statistics for the reduced model (the variables which lost significance in model 1 were removed and the model was then rerun). Despite the significance of each when entered into a model alone, when entered together only the psychological approach to successful ageing retained significance. Thus the biological (health) and social appraoches lost significance in this combined regression model. Mirroring the results of the single regressions of each approach to successful ageing, the only follow-up predictor variables which retained significance were health status, social support, social activity and self-rated active ageing.

**Table 3:** Full linear multiple regression of predictors of OPQOL+

Independent predictor variables	Unstandardised B	Standardised Beta	95% confidence interval	(2-tailed t-test)	P =
**Block 1**: R^2 ^change 0.254, p = 0.0001					

**Baseline approaches to successful ageing**					

SA biological (health)	0.198	0.013	--1.607-2.004	(0.217)	0.829

SA psychological	1.514	0.154	0.458-2.570	(2.827)	0.005

SA social	1.665	0.112	1.134-3.196	(2.145)	0.033

**Block 2**: R ^2 ^change 0.306, p = 0.0001					

**Follow-up health and social circumstances**:					

Self-rated health status, compared to others of same age	0.062	0.192	0.026-0.098	(3.406)	0.001

Number of people can turn to for comfort/support	0.524	0.172	0.232-0.816	(3.538)	0.001

Number of social activities	0.014	0.194	0.005-0.023	(2.971)	0.003

Self-rated active ageing	-4.645	-0.330	0.6.410--2.880	(-5.190)	0.001

**Block 3**: R^2 ^change 0.022, p = 0.070 ns					

Age	0.041	0.091	-0.182-2.264	(0.359)	0.720

Sex	1.520	0.054	-1.229-4.269	(1.091)	0.277

NS-SEC	4.686	0.130	1.271-8.101	(2.707)	0.007

Housing tenure (follow-up)	0.105	0.005	-1.898-2.109	(0.103)	0.918

Marital status (follow-up)	-0.031	-0.003	-1.191-1.130	(-0.052)	0.958

Constant	100.960				

R ^2^	0.560				

Adjusted R^2^	**0.545**				

Anova F statistic; p =	22.462; 0.0001				

**Table 4:** Reduced linear multiple regression of predictors of OPQOL

Independent predictor variables	Unstandardised B	Standardised Beta	95% confidence interval	(2-tailed t-test)	P =
**Block 1**: R^2 ^change 0.311, p = 0.0001					

**Baseline approaches to successful ageing**					

SA biological (health) (n. chronic conditions, ADL, GHQ-12 score)	0.039	0.053	1.273-3.352	(0.888)	0.376

SA psychological (for this regression self-efficacy and optimism only - minus GHQ items on self-worth and confidence as GHQ-12 included in biological model)	3.562	0.177	1.530-5.593	(3.462)	0.001

SA social (n. different social activities, n. areas supported in, face to face contact score)	1.155	0.066	-0.759-3.069	(1.192)	0.235

**Block 2**: R ^2 ^change 0.304, p = 0.0001					

**Follow-up health and social circumstances**:					

Self-rated health status, compared to others of same age	0.055	0.160	0.012-0.099	(2.529)	0.012

Physical functioning (activities of daily living/mobility score)	-0.011	-0.078	0.034-0.110	(-1.019)	0.310

Number of people can turn to for comfort/support	0.654	0.176	0.288-1.020	(3.525)	0.001

Number of social activities	0.013	0.169	0.003-0.024	(2.474)	0.014

Self-efficacy - problem solving	2.165	0.090	-0.384 - -4.714	(1.677)	0.095

Self-rated active ageing	-4.443	-0.313	-6.513 - -2.373	(-4.238)	0.001

**Block 3**: R^2 ^change 0.012, p = 0.397 ns					

Age	0.170 0.049		-0.212-0.552	(0.878)	0.381

Sex	0.261 0.009		-2.920-3.410	(0.878)	0.381

NS-SEC	-0.718 -0.095		1.481-0.045	(-1.857)	0.065

Housing tenure (follow-up)	0.887 0.038		-1.411-3.185	(0.762)	0.447

Marital status (follow-up)	-0.107 -0.008		-1.420-1.206	(0.161)	0.872

Constant	93.794				

R ^2^	0.627				

Adjusted R^2^	**0.595**				

Anova F statistic; p =	19.700; 0.0001				

Reduced model: significant variables only, with control variables, re-entered)

+ Baseline approaches to successful ageing with follow-up health and psychosocial variables, controlling for socio-demographic/economic characteristics (full model)

In the final multiple regression analysis of the relative predictive ability of independent biomedical (health), psychological and social approaches to successful ageing, only the baseline psychological approach (perceived self-efficacy and optimism) retained statistical significance. Socio-demographic and economic variables were not significant in the model. Follow-up self-rated health but not physical functioning was also significant, as were social support and participation and self-rated active ageing. Follow-up perceived problem solving abilities did not retain significance in the final regression model. The amount of explained variance in OPQOL scores by the variables entered was significant, and high at 60%.

## Discussion

The aim of the analyses presented here was to examine whether baseline appraoches to successful ageing predicted QoL, at follow-up 7-8 years on. Biomedical (health) approaches to successful ageing are the most widely used and published. Promotion of QoL tends to be framed in conventional medical terms of mental and physical health. However, the longitudinal results presented here caution against over-reliance on sole biomedical (health) approaches to successful ageing. In the multiple regression analyses of the relative predictive ability of independent biomedical (health), psychological and social approaches to successful ageing, only the baseline psychological approach (perceived self-efficacy and optimism) retained significance. While baseline analyses found that the multidisciplinary lay approach (which incorporated the other approaches) was the strongest predictor of a global quality of life (measured using a single, global item question), these earlier analyses examined each approach in separate regressions (12). The results presented here differed from the baseline analyses, as, for this paper, each approach was entered into the same regression analysis (hence overlapping approaches had to be excluded as they were not independent - the broader biological (health and social engagement) and the lay approach).

Huppert's (2008) [5] review of mental capital and well-being emphasised the importance of the influence early environmental factors on mental well-being, as well as external circumstances, but concluded that individuals' actions and attitudes may have a greater influence. Hence, enhancement of well-being requires interventions to encourage positive attitudes and behaviours over the life course. However, evidence indicates that self-efficacy and reliance can also be nurtured in later life [24,25]. This study of influences on QoL outcomes supports the literature on the importance of building up one's psychological resources in order to cope effectively with the challenges of ageing, given that it is difficult for very elderly people, who are frail, to function physically at optimal levels and retain high levels of activity [16,26]. The narrowness of a dichotomous approach to having successfully aged (on all indicators) or not was also illustrated by the minorities of people who had all good scores within each approach. This limited approach can stigmatise and marginalise older people with disabilities [27]. A continuous approach to conceptualisation and measurement is preferable. In single model regression analyses the dichotomised biological (health) approach was also less sensitive to QoL outcomes.

Policy makers aiming to promote wellbeing, successful ageing and QoL in ageing populations should consider people's psychological resources, rather than only their health, functional, activity levels or social circumstances (which deserve attention for other reasons). QoL is often seen as an outcome of service activities, such as encouraging uptake of preventive care, or modifying lifestyles. For example, the US Centre for Disease Control focuses its efforts on improving QoL by promoting healthy lifestyle, behaviors, increasing the use of clinical preventive services, addressing cognitive impairment, addressing issues related to mental health and provide education on decision making related to end-of-life planning (http://cdc.gov/chronicdisease/resources/publications/aag/aging.htm - link valid 03-03-2011). Promoting psychological resources is crucial for optimising both ageing well or successfully, and enhancing the quality of later life, enabling older people to feel confident in living in their own homes, and with wider benefits to society.

The limitation of the study must also be acknowledged. The survey used statistically robust sampling methods, and the response rates were fairly good in an era of declining response to surveys. Sample attrition is inevitable in longitudinal surveys, especially in older sample members, where the most vulnerable and ill members of the sample will have died or dropped out, leaving the healthiest sample members. In a follow-up study of older people there is inevitably a healthy survivor effect among the respondents. Hence the results relate to a sample of survivors, and cannot necessarily be generalised across older populations. While the characteristics of respondents were comparable with population estimates from the last census, non-response was still a potential source of bias. The respondents who had died since baseline were more likely to be older, male and to have worse baseline heath status. Follow-up health status, along with number of supporters and social activities, and self-rated active ageing, also significantly predicted QoL at follow-up. Thus the longitudinal sample bias towards healthy survivors is likely to underestimate these results.

There was some positivity bias in ratings of QOL and successful ageing. This was not unexpected. Lawton's (2001) [28] theory of emotion-regulation argued that older people are more likely than younger people to regulate affect, and minimise the negative, while maximising the positive. There is some supporting evidence for this theory, although results are inconsistent.

In conclusion, our findings suggest that healthy ageing is not simply about physical or mental health maintenance, but rather about maximising psychological resources, namely self-efficacy and resilience. Increasing use of preventive care, better medical management of morbidity and changing lifestyles in older people may have beneficial effects on wider health and longevity, but may not improve their quality of life. Adding years to life and life to years may require two distinct and different approaches, one physical and the other psychological. A psychological approach includes perceived self-efficacy, self-esteem and self worth, confidence, optimism, purpose in life, coping, facing up to problems and overcoming difficulties. Only the psychological approach to successful ageing independently predicted quality of life at follow-up.

## Abbreviations

OPQOL: Older People's Quality of Life Questionnaire; QOL: quality of life

## Competing and financial interests

The authors declare that they have no competing interests.

## Authors' contributions

AB carried out the statistical analyses, and wrote the initial draft of this paper. SI contributed significantly to developing the idea for the study, and to subsequent drafts of this paper; he had access to the data. AB conceived of the study, had full access to all the data in the study and takes responsibility for the integrity of the data and the accuracy of the analyses. Both authors have read and approved the submitted manuscript.
